# Preparation of Dispersed Copper(II) Oxide Nanosuspensions as Precursor for Femtosecond Reductive Laser Sintering by High-Energy Ball Milling

**DOI:** 10.3390/nano13192693

**Published:** 2023-10-02

**Authors:** Kay Bischoff, Cemal Esen, Ralf Hellmann

**Affiliations:** 1 Applied Laser and Photonics Group, University of Applied Sciences Aschaffenburg, Würzburger Straße 45, 63743 Aschaffenburg, Germany; 2 Applied Laser Technologies, Ruhr University Bochum, Universitätsstraße 150, 44801 Bochum, Germany; esen@lat.rub.de

**Keywords:** reductive laser sintering, high-energy ball milling, laser diffraction analysis, nanoparticle, laser digital patterning, copper electrode, CuO, precursor, nanosuspension, dispersion

## Abstract

This contribution demonstrates and discusses the preparation of finely dispersed copper(II) oxide nanosuspensions as precursors for reductive laser sintering (RLS). Since the presence of agglomerates interferes with the various RLS sub-processes, fine dispersion is required, and oversized particles must be identified by a measurement methodology. Aside from the established method of scanning electron microscopy for imaging individual dried particles, this work applies the holistic and statistically more significant laser diffraction in combination with dynamic image analysis in wet dispersion. In addition to direct ultrasonic homogenization, high-energy ball milling is introduced for RLS, to produce stable nanosuspensions with a high fine fraction, and, above all, the absence of oversize particles. Whereas ultrasonic dispersion stagnates at particle sizes between 500 nm and 20 μm, even after 8 h, milled suspension contains a high proportion of finest particles with diameters below 100 nm, no agglomerates larger than 1 μm and a trimodal particle size distribution with the median at 50 nm already, after 100 min of milling. The precursor layers produced by doctor blade coating are examined for their quality by laser scanning microscopy. The surface roughness of such a dry film can be reduced from 1.26 μm to 88 nm by milling. Finally, the novel precursor is used for femtosecond RLS, to produce homogeneous, high-quality copper layers with a sheet resistance of 0.28Ω/sq and a copper mass concentration of 94.2%.

## 1. Introduction

Printed electronics, combining conventional printing technologies with next-generation electronic device fabrication, have attracted increasing attention in the field of materials science and technology [1,2,3]. In particular, laser direct writing (also known as laser digital patterning) with conductive ink is becoming increasingly popular as a technique for printed electronics. This maskless process does not require vacuum, lithography or patterning processes, and can be customized by simply adjusting the laser pattern. It offers excellent suitability for rapid prototyping [4,5,6]. In polymer metallization, selective electroless plating exists, but it requires initial doping or surface patterning of the polymer [7,8,9].

For laser direct writing, the development of processes using noble metal particles was initially advanced; however, the use of the more available, less expensive and excellently conductive copper (Cu) became increasingly prevalent [2,10,11]. Due to its low oxidation potential, the sintering of metallic Cu particles requires special precautions, such as inert atmospheres and special storage of the particles. One solution provides the use of a stable precursor (PC) in the form of salts [12,13,14,15], complexes [16] or oxide nanoparticles (NPs), such as CuO or Cu_2_O [17,18], in reductive laser sintering (RLS). Modification of the copper layer has also been demonstrated for identification of disease markers [19]. Aside from Cu, the RLS approach has also been demonstrated with nickel [20,21] and cobalt [22].

Kang et al. [23] first demonstrated RLS with CuO NPs as a process that can be performed with a stable PC at an ambient atmosphere, which triggered a high degree of research interest from various research groups. A substrate is first coated with the PC. Then, energy is selectively applied to the layer, using a laser to initiate the thermochemical reduction process that converts the oxide to metallic Cu and sinters it, leaving a conductive Cu layer after the residual PC is rinsed off. Later, Arakane et al. [24] first demonstrated the use of femtosecond pulsed lasers, which has continued in many research papers, due to the precise control of the energy deposited into the PC, as well as the low thermal load on the substrate [25,26,27,28,29,30]. The advantages of CuO over other PCs include its high absorption, as well as short process times, so it has also become popular for comparable processes, such as the intense pulsed light process [31,32,33]. In a variety of studies, CuO-based RLS has been used to electrify not only glass [23,24,27] but also polymers, including highly heat-sensitive ones, such as polyamide (PI) [23,34,35], poly(dimethylsiloxane) (PDMS) [36], polyethylene terephthalates (PET) [37] and cyclic olefin copolymers (COC) [28,29]. Thus, applications such as humidity sensors [38], transparent touch screen panels [37], optoelectronic systems [28,29], p- and n-type thermoelectric micropatterns [26], 3D thermal flow sensors [39], micro-temperature sensors [40] and micro-heaters [28] have been demonstrated and highlight the enormous potential of this technology.

**Figure 1:**
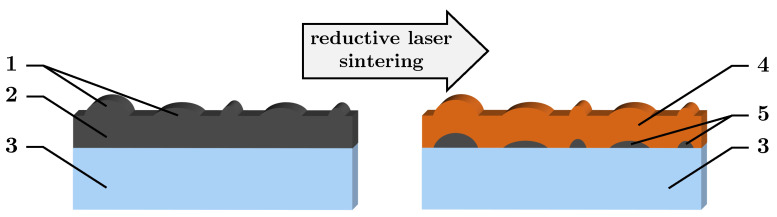
Schematic illustration of RLS on PC layers containing big agglomerates: 1. large agglomerates at the surface; 2. dry PC layer; 3. substrate; 4. undefined Cu layer; 5. residual PC.

For a controllable process in RLS, a homogeneous, defect-free PC layer consisting of ultrafine NPs is required. If the layer contains agglomerates that significantly exceed its thickness and the depth of reduction in RLS, it must be assumed, as shown in Figure 1, that an inhomogeneous Cu layer is formed. At the positions of the agglomerates, reduction cannot take place all the way to the substrate, leaving areas of unreduced PC. These can become detached during the rinsing process, eroding the Cu layer and compromising substrate adhesion. Binh Nam et al. [37] compared commercial ultrasonically dispersed NPs to custom-synthesized particles, and they showed that the commercial ones have disadvantages. Finally, smaller NPs exhibit lower melting temperature and higher reactivity [41]. A PC coating with larger particles provides higher surface roughness of subsequent Cu layers, which might promote oxidation. In addition, the smaller the particles in the PC, the more defined the edges of the resulting Cu traces. Furthermore, in the context of RLS with Cu_2_O NPs, it has been shown that larger particles result in higher electrical resistivity [17].

To produce nanosuspensions, in addition to the one-step method, whereby the NPs are formed directly in the subsequent suspension, the two-step method exists. Here (mostly commercial) NPs in powder form are dispersed in the liquid. The latter has been proven to be more economical, due to the high availability of the powders, and it has consequently prevailed [42,43]. In previous RLS research, mostly commercial CuO NP powders were used for PC preparation, but only superficial investigations were performed on how dispersion must be performed or what particle sizes are achieved in a real-liquid PC. It is evident that, apart from the few works involving custom synthesis of the particles, only ultrasonication has been used to disperse commercial NPs, or the dispersion methodology is only marginally described. While research exists on the successful ultrasonic dispersion of CuO NPs, particularly the characterization of this process, this work is limited to solutions with very low particle concentrations (<2 wt%) [44]. RLS, by contrast, uses highly concentrated nanosuspensions with particle concentrations up to 60 wt%, for good coatability [23,24,35].

In terms of NP production, in recent decades, mechanical milling has become an established technique via the top-down method [45,46,47], but also to disperse agglomerated NPs by high-energy ball milling (HEBM), e.g., CuO [48,49,50,51]. Ettefaghi et al. previously found that ball milling is preferable to ultrasonication for high-viscosity nanofluids [52].

A variety of methods are available to determine particle shape and size distribution [53]. Transmission electron microscopy and scanning electron microscopy (SEM), which have been primarily used in RLS research to date, are very suitable for assessing primary particle size and shape, but do not reflect the actual statistical situation in wet fluid [42,54]. Dynamic light scattering (DLS) can provide high resolution of the particle size distribution, especially for particle sizes in the sub-100 nanometer range, but larger agglomerates are barely measurable if they sediment and leave the focal voxel too fast [55]. Laser diffraction measurement is less affected by this issue, because the measuring fluid is permanently pumped, and larger and heavier agglomerates can be measured, so that the progress of a dispersion can be observed [56]. The measured particles are illuminated with one or more lasers, so that the light is diffracted by the particles and produces scattering patterns on a detector, which gives information about the particle size distribution. In particular, the use of short-wavelength blue lasers allows particle sizes down to 10 nm to be measured accurately and efficiently [57].

Against this broad background, this work introduces the dispersion of a highly filled, commercial CuO nanoparticle-based precursor for reductive laser sintering by high-energy ball milling and compares it to direct sonotrode-based ultrasonic dispersion. Beyond our previous publications on RLS [28,29], here we focus on PC production, with the targeted particle size below 1 μm. To validate the achieved particle sizes, we also show the particle size distribution in the liquid state of the PC, by laser diffraction analysis combined with dynamic imaging, in addition to SEM analyses. The effects of dispersion on the resulting PC films, in terms of surface quality and film thickness homogeneity, as well as the resulting femtosecond RLS Cu films, are also presented.

## 2. Materials and Methods

### 2.1. Chemicals and Materials

Polyvinylpyrrolidone (PVP) K12 (2000– 3000 g/mol), ethylene glycol (EG) (>99%) and Ethanol (>99.8%) were purchased from Carl Roth (Karlsruhe, Germany). CuO-NP (40– 80 nm) were optained from Iolitec (Heilbronn, Germany). COC substrates (TOPAS 6017S-04) were purchased from TOPAS Advanced Polymers (Raunheim, Germany).

### 2.2. Preparation of the Nanosuspension

To prepare the CuO nanosuspension by the two-step method, PVP was dissolved in EG by indirect ultrasonication and stirring for 1 h, and then added to powdered CuO-NPs through a syringe filter and stirred to a homogeneous high-viscosity paste. It should be mentioned that the observations made in this work were also made with particles from other batches and from another manufacturer (Carl Roth, Karlsruhe, Germany). The concentrations corresponded to the mass ratio 60:27:13 wt% (CuO:EG:PVP), which is widely used in the literature [23,24,35]. Ethanol was added, to temporarily lower the viscosity, so as to facilitate dispersion. Ethanol does not form an acetropic mixture with EG (as measured by Abbe refractometry), and it was gently distilled out after dispersion by means of a rotary evaporator (RV 3, IKA, Staufen, Germany) at 40 ${}^{\circ }C$ and mild vacuum. After cooling the liquid PC, the vacuum was increased (2 mbar), for degassing.

For ultrasonic dispersion, the sonotrode (2 or 7 mm) of the ultrasonic homogenizer (UP200St, Hielscher, Teltow, Germany, 26 kHz, 200 W) was immersed directly into the nanosuspension. The viscosity was reduced until cavities were formed. Homogeneous sonication of the fluid was achieved by off-center positioning of the sonotrodes in the rotating beaker. Sequential sonication (10 s on– 25 s off) avoided overheating of the fluid, which also prevented re-agglomeration of the NPs. For the HEBM dispersion, a high-energy ball mill (MM 500 nano, Retsch, Haan, Germany) with a frequency of 35 Hz, an amplitude of 20 mm and an acceleration of 50.3 g was utilized. Using the conventional ball-to-particle-mass ratio of 10:1, 18.33 g CuO suspension with 110 g zirconia grinding balls (500 μm diameter) were added to the 50 mL grinding jars (zirconia). Ethanol was added until the viscosity was reduced enough for efficient milling (10 mL). Sequential milling (10 min @ 35 Hz– 10 min @ 7 Hz) was used, to reduce thermally induced re-agglomeration. The dispersed suspension was separated from the balls by rinsing with ethanol and a vibrating sieve.

### 2.3. Generation of Copper Layers by Femtosecond Reductive Laser Sintering

**Figure 2:**
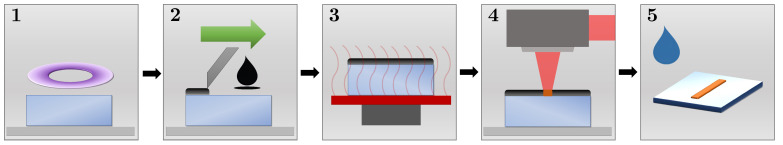
Schematic illustration of femtosecond reductive laser sintering using CuO PC with: 1. plasma activation of specimen; 2. doctor blade coating of CuO PC; 3. PC drying on hot plate; 4. selective laser processing of PC layer; 5. rinsing of residual unprocessed PC.

The process chain of femtosecond RLS consists of the following steps, as shown in Figure 2. COC substrates were oxygen plasma activated (Pico, Diener, Ebhausen, Germany), to ensure wettability (40 kHz, 0.2 mbar, 180 W, 3 min). After the PC was prepared and adjusted, by setting the viscosity for good coatability by adding EG dropwise, it was applied to a 50 × 50 mm${}^{2}$ COC substrate and coated by the doctor blade method (ZAA 3000, Proceq, Schwerzenbach, Switzerland). The height-adjustable applicator was moved at a speed of 10 mm/s, resulting in dry film thicknesses of approximately 20 μm. The coating was dried on a hot plate at 70 ${}^{\circ }C$ for 30 min. RLS was performed, using a 10 W ultrashort pulsed laser (Pharos-10-600-PP, Light Conversion, Vilnius, Lithuania). The laser beam—with a wavelength of 1030 nm, a pulse duration of 220 fs and an adjustable pulse frequency up to 610 kHz—was scanned on the specimen, with a galvanometer scanner (Rhothor AR800, Newson, Dendermonde, Belgium) equipped with a telecentric f-theta lens (focal length 100 mm), resulting in a focal spot size of about 95 μm under defocus of 2 mm. The PC layer was exposed, with a repetition rate of 100 kHz. The two-dimensional Cu layers consisted of a hatched structure with an overlap of 50% with respect to the width of a single Cu track. After the laser process, the residual unsintered PC can be successively rinsed with water and EG. A comprehensive description of the RLS process is given by the authors and others, elsewhere [28,29].

### 2.4. Evaluation Methods

The particle size distribution within the nanosuspension was measured, using a laser diffraction particle size analyzer equipped with two blue lasers and one red laser, in combination with dynamic image analysis (SYNC 2B1R DIA, Microtrac, Haan, Germany) and the FlowSync module in wet dispersion. In this manner, a size range from 10 nm to 2 mm could be covered, and small amounts of oversized particles could be detected in addition to the fine content. The sample was added undiluted to the measuring reservoir, which was filled with deionized water, to avoid sedimentation of large particles during dilution prior to addition. A mild ultrasonification could be applied to the system prior to measurement, to separate newly formed flocculates. The particle size distribution was calculated automatically, using the Flex 12 software (Microtrac), assuming non-spherical opaque particles (n = 2.63) from the light scattering pattern, as well as the image acquisitions of the analyzer. The measurement was performed in triplicate.

To visualize the particles and agglomerates, the nanosuspension highly diluted in ethanol was dried on a plasma activated glass slide and coated with a conductive gold layer, by sputtering. Images were obtained using an SEM (MAIA3, Tescan, Brno, Czech Republic) at an accelerating voltage of 8 kV in the ultrahigh-resolution mode. Size measurement of the SEM images was performed using the software Gwyddion (Czech Metrology Institute, Jihlava, Czech Republic). Optical inspection of the PC surface and Cu layers was performed, using a digital microscope (DMV6, Leica, Wetzlar, Germany), in addition to SEM. The surface quality of the dried PC layers was evaluated, using a laser scanning microscope (LSM) (VK-X200, Keyence, Osaka, Japan). The average surface roughness Sa and peak-to-valley height Sz were validated in areas of 285 × 214 μm${}^{2}$, respectively.

The material composition of the Cu films produced was measured, using an energy-dispersive X-ray spectroscopy sensor (AZtec, Oxford Instruments, Abingdon, United Kingdom) of the SEM on areas of 500 × 500 μm${}^{2}$ at an accelerating voltage of 30 kV. The measurement and calculation of the electrical surface resistance was performed by means of a 4-tip measuring instrument (Loresta GX, Mitsubishi Chemical Analytech, Tokyo, Japan) equipped with a probe with square arranged measuring tips (QP2, Mitsubishi Chemical Analytech, Tokyo, Japan). In triplicate, 10 × 10 mm${}^{2}$ Cu areas were measured with a central attachment of the measuring probe, in two orientations.

## 3. Results and Discussion

### 3.1. Undispersed CuO Nanosuspension

**Figure 3:**
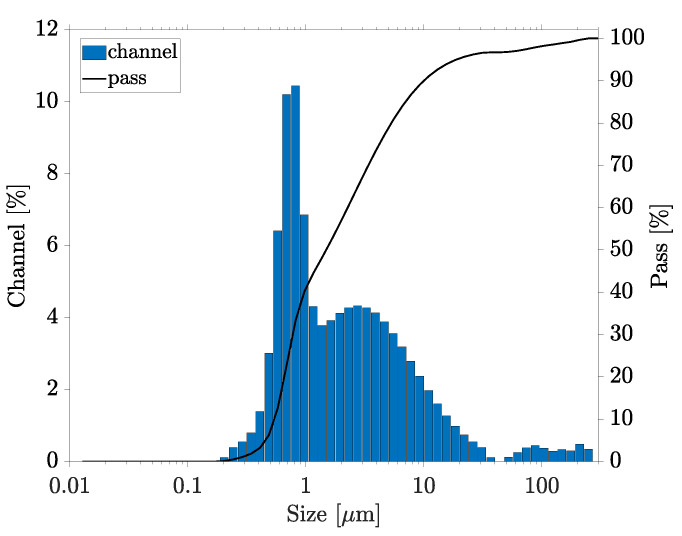
Particle size distribution in the PC without dispersion, measured by particle size analyzer.

First, the behavior of the PC without high-energy dispersion was investigated by indirect sonication in an ultrasonic cleaner for 15 min with continuous stirring. The particle size measurement showed a very large fraction of particles from several hundred nanometers up to more than 100 μm (Figure 3). No proportion of near-primary particle size below 100 nm could be measured, while a strong oversize was detected. The percentiles were measured as D10 = 0.74 μm, D50 = 3.68 μm and D90 = 61.23 μm. Based on dynamic image analysis, irregularly shaped agglomerates could be visualized. The application of ultrasound through the flow system of the particle size analyzer (100% for 1 min) showed only a slight dispersion.

**Figure 4:**
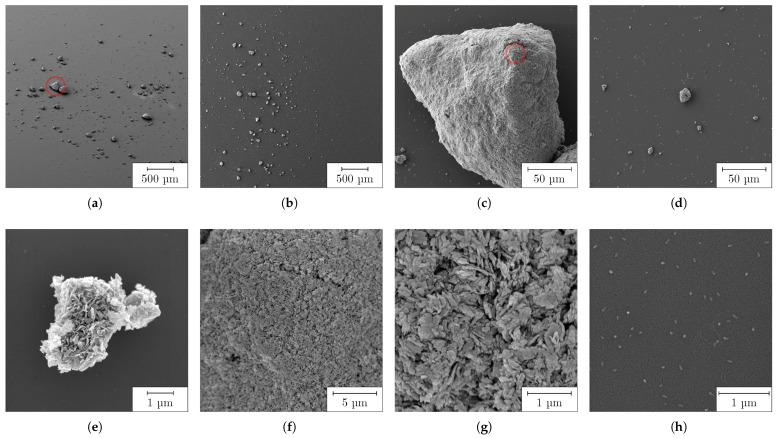
Undispersed CuO particles visualized by SEM: (**a**,**b**) overview; (**c**) large agglomerate from (**a**); (**d**,**e**) medium-sized agglomerates; (**f**,**g**) detailed, enlarged view of large agglomerate from (**c**); (**h**) individual primary particles.

The analysis of the particles by SEM is shown in Figure 4. Large agglomerates, mostly measuring tens of micrometers, were already visible in the overview images (a) and (b). Small agglomerates in the range of a few micrometers (d and e) and up to 210 μm (c) were measured. Further enlargement of the agglomerates (f and g) showed that they were partially composed of plate-like NPs, which were about 100 nm to 600 nm wide and 25 nm to 70 nm thin. In addition, there were isolated, almost spherical, particles in the size range of tens of nanometers (h), as well as their accumulations to islands (d). The SEM results very accurately reflected the previous measurement of liquid PC and clearly showed that the large particles measured there were agglomerates or aggregates consisting of individual NPs. Although individual primary particles could be imaged here, it can be assumed that they represented a minority in the volume of the PC, so that they could not be detected by laser diffraction. It should be emphasized, however, that although the SEM measurements provided very good information about the shape of the particles and their size ranges, nevertheless we did not derive any statistical size distributions from them.

**Figure 5:**
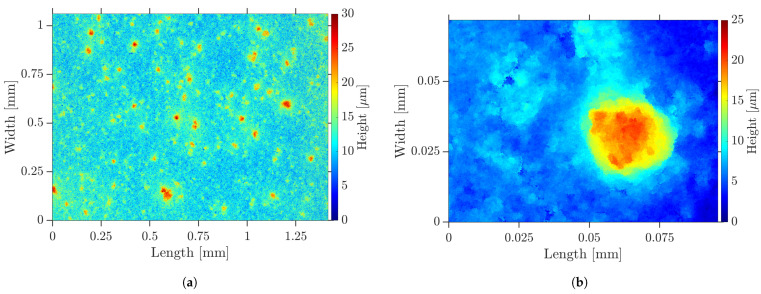
Dry undispersed PC layer—topography visualized by LSM: (**a**) overview; (**b**) agglomerate.

**Figure 6:**
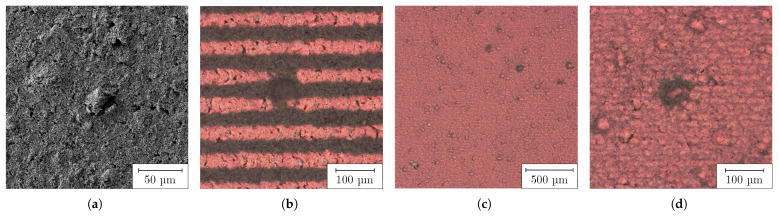
Dry undispersed PC layer: (**a**) agglomerate on unprocessed PC visualized by SEM; (**b**) RLS-generated Cu lines with agglomerate-induced defects; (**c**) 2D Cu layers with agglomerate-induced defects (30 mm/s at 1 μJ); (**d**) detailed view of (**c**), visualized by optical microscopy.

When the undispersed PC was coated on a COC substrate, the inhomogeneous surface shown in Figure 5 by LSM and in Figure 6a by SEM was formed. It was interspersed with defects, such as agglomerates, pores and cracks. The pores could also be observed by transmitted light microscopy. These defects were assumed to form on the flanks of the agglomerates during drying. The surface roughness measured 1.26 μm and the peak-to-valley height was 21.58 μm. When the fs RLS was performed on such a PC layer, the defects propagated into the resulting Cu layer, as shown in Figure 6b for lines and (c) and (d) for 2D layers. In locations where large agglomerates were present, both lines and areas were not completely reduced, so that electrical conductivity could not be present. On the steep flanks of the agglomerates, there was less absorption of the laser beam, so the temperature required for reduction could not be reached. In addition, the large agglomerates were less reactive and could only come into contact with the reducing agent from the outside. As a result, the chemical process would possibly not take place completely to their core.

In areas where the agglomerates protruded from the surface level, it must be assumed that no RLS could take place down the substrate surface, and thus the conductivity of the resulting Cu layer was severely impaired. Therefore, dispersion was urgently needed, to obtain homogeneous Cu layers in the RLS that were not underwashed and delaminated in the cleaning step.

### 3.2. Ultrasonic Dispersion of the CuO Nanosuspension

**Figure 7:**
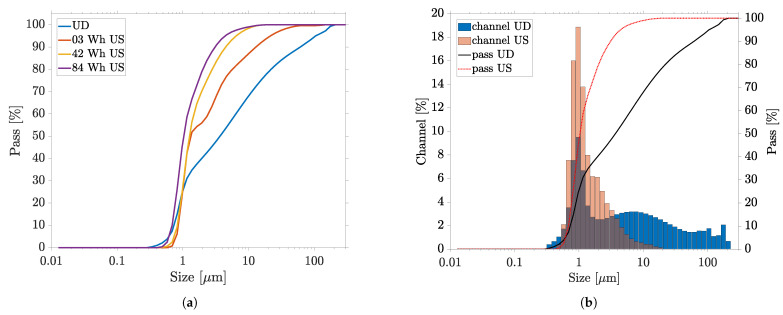
Evolution of particle size distribution by direct ultrasonic dispersion (US) measured by laser diffraction particle size analyzer: (**a**) dispersion course; (**b**) comparison of undispersed (UD) masterbatch—final fineness achieved.

The ultrasonication was performed in two steps with two sonotrodes, by applying an energy of 42 Wh each to the PC. The progress of the dispersion was studied, by measuring the particles of fluid extracted in the intermediate stage, and is shown in Figure 7a. First, a 2 mm-tip-diameter sonotrode was used at 100% amplitude (210 μm—equivalent to 35 W effective power). The first particle size measurement after 20 min and an energy of 3 Wh indicated that the dispersion of the agglomerates shown in the previous section had occurred. The sonication continued until the introduced energy of 42 Wh was reached after the total process time of 6:20 h. The particle size measurement showed that the particles continued to decrease in size.

A 7 mm sonotrode was used as another configuration, where a smaller amplitude (175 μm) prevailed but a larger power (74 W) was deposited into the fluid. After introducing additional ultrasonic energy of 42 Wh within 2:20 h, the particle measurement showed only a slight difference. The final percentiles were measured as D10 = 0.74 μm, D50 = 1.06 μm and D90 = 3.08 μm. Although large agglomerates were further reduced in size by prolonged ultrasonication, the dispersion stagnated in the fine fraction. It was evident that the fine fraction of the particles was minimally enlarged, which can be attributed to the thermal treatment (8 h at 80 ${}^{\circ }C$) of the PC. The median D50 remained almost unchanged, indicating that the aggregates corresponded to this size. The high filling level of the nanosuspension may also have been partly responsible for the lower efficiency of the ultrasonic dispersion, compared to work with highly diluted NP solutions with less than 2 wt% particle content [44]. The ultrasound-induced cavitations were hindered in their propagation, and less turbulence was created in the fluid. To circumvent this aspect, a much more complex and expensive system technology would be required. The final particle size distribution was compared to that of the initial fluid in Figure 7b. A monomodal but broad particle size distribution, between about 500 nm and 20 μm, was obtained. Although the oversize was reduced to about 10%, however, no particles in the primary particle size range were detectable. Since the goal of achieving the primary particle sizes and greatly reducing the oversize could not be achieved, even after prolonged sonication, further investigation of the PC was abandoned.

Although ultrasonic dispersion has been the most commonly used dispersion method in previous research on RLS, and although we obtained high amplitudes of up to 210 μm, sufficient dispersion could not be achieved, even after prolonged ultrasonication of the PC. While the most disturbing agglomerates could be shrunk to 10% of their size, the process stagnated in the sub-micron range of particle size, and no primary particles were obtained. This shows that, in addition to agglomerates, there must be aggregates that require higher energy than ultrasonication can provide, to break them up. We conclude that direct ultrasonic dispersion can approximate the main problems of the RLS method in the presence of highly agglomerated particles. However, the process is accompanied by a long process time, high thermal stress and high sonotrode wear, leading to Ti contamination from the sonotrode.

### 3.3. High-Energy Ball Milling of CuO Nanosuspension

#### 3.3.1. Characterization of the Milling Process

**Figure 8:**
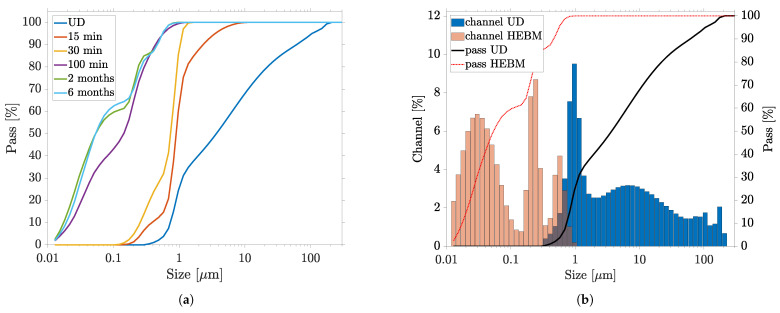
Evolution of particle size distribution by HEBM dispersion and long-term stability measured by laser diffraction particle size analyzer: (**a**) dispersion course with milling time in min, and measurements after 2 and 6 months; (**b**) comparison of undispersed (UD) PC—final fineness achieved (measurement after 2 months).

**Figure 9:**
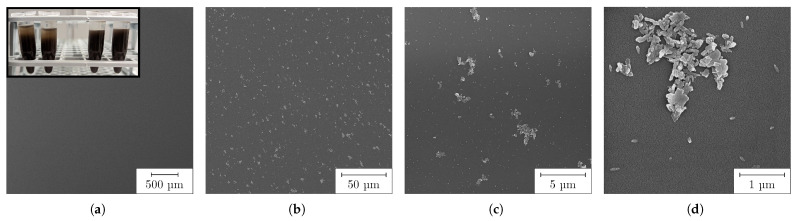
HEBM-dispersed CuO particles: (**a**,**b**) overview; inset: sedimentation test—HEBM (left), undispersed (right); (**c**) islands of fine particles formed during drying, isolated particles and small residual agglomerates; (**d**) detailed, enlarged view of particle islands and individual primary particles.

To investigate the effect of milling time on particle size distribution, HEBM of 100 min was employed. To avoid contamination of the PC by ZrO, a longer milling time was omitted. First, the HEBM dispersion aimed to be characterized by interrupting the sequential HEBM after 15 and 30 min of milling time, for particle size measurement. The 100 min-milled PC was also remeasured after storage of 2 and 6 months. Figure 8a shows how the particle size distribution behaved with increasing milling and storage time. It is evident that the total particle size substantially decreased with increasing milling time. Especially within the first 15 min, a drastic breaking up of the large agglomerates was observed. Only after complete the HEBM were sub- 100 nm particles measured. The percentiles change with milling time: D10 decreased from 0.738 μm to 0.018 μm, D50 decreased from 3.68 μm to 0.05 μm and D90 decreased from 61.23 μm to 0.56 μm for the PC measured after 2 months. The final particle size distribution measured after 2 months was compared to that of the initial fluid in Figure 8b. A trimodal distribution with modes of about 35 nm, 210 nm and 510 nm was obtained. On the one hand, the oversize was completely reduced to particles below 1 μm and, on the other hand, the dispersion was achieved down to the primary particle size and even below. Compared to ultrasonic dispersion, the HEBM fragmented large agglomerates and aggregates significantly faster and more efficiently. Already, after a milling time of 15 min, the percentiles of the total ultrasonic dispersion were undercut. Similar to ultrasonication, the large particles appeared to be more easily separated than the particles in the 1 μm size range. After the HEBM of the oversized particles, fine fraction of the nanosuspension was successively produced and was further reduced in size, until the trimodal distribution of particle size was finally achieved after 100 min. A similar result could be obtained by milling the PC without PVP content, showing that the particles present could be stabilized even after milling. Furthermore, comparative sedimentation tests (strong dilution of the PC in ethanol) showed that the undispersed PC started to sediment after 6 h, while the PC dispersed by the HEBM remained unchanged (see Figure 9 inset).

The particle size distribution measured by means of the laser diffraction method showed a high proportion of particles in the sub 50 nm range, where the resolution limit of this technology was reached. The slight increase of the fine fraction in the particle distribution while storing led to the conclusion that, immediately after milling, particles smaller than 10 nm were present, which were not recorded by laser diffraction. Only when these particles agglomerated over time did they affect the measurement result, so that the particle sizes appeared to decrease during storage. Regarding RLS, we conclude that laser diffraction combined with dynamic image analysis should nevertheless be the preferred method for measuring particle size distribution, with respect to the presence of agglomerates and to characterization of the dispersion process. In future studies, PCs produced by HEBM can be additionally examined by DLS, in order to provide a more accurate statement of the fine particle fraction.

The individual particles of the HEBM-dispersed PC stored for 2 months are shown by SEM in Figure 9. Again, there was a strong reduction in particle size compared to the undispersed PC (see Figure 4). The overview image (a) shows a homogeneous surface, free of large agglomerates. Only on further magnification (b) are islands of NPs and small agglomerates visible, which presumably accumulated during the drying process. The detailed images in (c) and (d) confirm that these appear to have been the nanoplates and rods as daughter particles of the undispersed agglomerates from Figure 4. These were present in a size range from 95 nm to 500 nm. In addition to these plates and rods, there were also very fine, spherical NPs, similar to those shown in Figure 4h, with an average diameter of about 80 nm (cf. Figure 9d). These observations confirmed the laser diffraction measurements and trimodality very well. We assumed that the two measured modes at 210 nm and 510 nm could be assigned to the two major axes of the nanoplates, while the mode at 35 nm reflected the finest obstructed particles and minor axes of the plates.

We concluded from the two measurement methods that the large agglomerates initially separated into individual nanoplates within a few minutes of milling, so that the proportion of oversize particles was significantly reduced. Only when the HEBM continued to apply high energy did these plates break and separate into particles in the sub- 100 nm size range. In particular, the high collision and friction forces of the mill used, with very high acceleration and frequency, ensured efficient and fine milling. It can be assumed that an even finer particle size distribution could be achieved by further HEBM. Milling times of several hours have been reported in the literature [49,51]. Moreover, the largest particles of the suspension were many times smaller than the target thickness of the subsequent PC layer of about 20 μm for the RLS. We concluded that no interfering defects occurred here during the coating (grooves in the layer) or in the further course of the RLS process.

The laboratory scale tests performed in this work were able to produce about 30 g of PC by parallel HEBM in two jars, which is sufficient in a research context or within the scope of rapid prototyping or to coat a large number of substrates. The utilized mill can also be equipped with larger jars and, in general, such processes are well transferable to larger batches, paving the way to industrial process scales.

#### 3.3.2. Reductive Laser Sintering with the Milled Precursor

**Figure 10:**
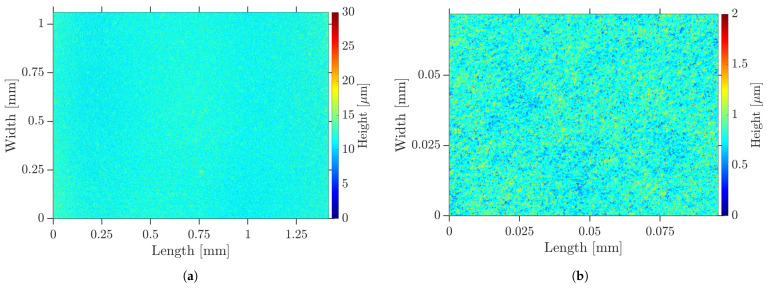
Surface topography of dry HEBM-dispersed PC layer on COC visualized by LSM: (**a**) overview; (**b**) detail.

**Figure 11:**
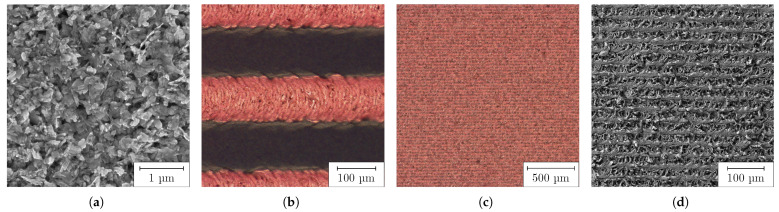
Dry HEBM-dispersed PC layer on COC: (**a**) unprocessed PC visualized by SEM; (**b**) RLS-generated Cu lines (30 mm/s at 3.5 μJ); (**c**) RLS-generated 2D Cu layers (150 mm/s at 3.5 μJ), visualized by optical microscopy; (**d**) detailed view of (**c**), visualized by SEM.

After fabrication of the PC by HEBM, samples were coated for RLS. The dispersed PC allowed a significantly higher dilution than the undispersed one, with respect to the wetting of the plasma-activated COC surface, so that a better adhesion of the resulting Cu layer could be assumed. The dry films appeared as illustrated in Figure 10 by LSM as a smooth and homogeneous surface of uniform thickness. There were no big agglomerates, pores, protrusions or cracks caused by agglomerates. The average surface roughness was measured to 88 nm and the peak-to-valley height was determined as 2.76 μm. The surface roughness Sa was reduced by a factor of 14 compared to the undispersed PC. The SEM image in Figure 11a shows a similar behavior. The previously described nanoplates and rods can be identified.

The Cu layers produced by means of RLS appeared highly homogeneous, with a sheet resistivity of 0.278 Ω/sq when the PC was generated by HEBM. Both the individual Cu lines (b), produced with 30 mm/s at 3.5 μJ, and their hatched areas ((c) and (d)), fabricated using 150 mm/s and 3.5 μJ, are shown in Figure 11. In contrast to the Cu structures fabricated with undispersed PC, there were no discontinuities or other defects. The individual Cu lines were linear, with sharply defined edges. Using energy-dispersive X-ray spectroscopy, the elemental mass composition of the Cu surfaces produced was determined to comprise Cu: 94.2%, carbon: 3.5%, oxygen: 2.2% and zirconium: 0.1%. The result shows that during the milling process there was only minor contamination of the PC by zirconia abrasion, and at the same time Cu layers with high Cu concentration were produced by RLS.

Due to the laser process and the associated abrupt decompensation of the PVP within the PC film, nanoporosity was generated, which—in addition to residual PVP and CuO or Cu_2_O formed by re-oxidation—could reduce the conductivity of the resulting Cu layer. The achieved low sheet resistance was comparable to previous research results and could serve a variety of applications [28,30]. The purpose here is merely to show that the RLS process can be well implemented, using a PC dispersed by HEBM, but this work does not claim to optimize the Cu properties. Also, it is expected that smoother PC surfaces have different absorption characteristics, with respect to the laser beam, and thus, that the parameters found in previous publications [28] cannot be fully transferred. This leads to the fact that the resulting properties of the Cu structures, such as line widths, are barely comparable. Further research on this aspect is needed, regarding the exact formulation of the PC, as well as an adjustment of the laser and process parameters to RLS with CuO PC. In addition, it has been shown, in the context of other PC types, that the thickness of the PC also has an influence on the resulting electrical resistance [58]. To date, no consistent determination of an ideal particle size and shape for CuO-based RLS has been made. Chiu et. al have already shown, in the context of photonic sintering, that a bimodal particle size distribution is advantageous for RLS, where the smaller embedded particles fuse the larger ones [35]. We therefore conclude that the trimodality of the particle size distribution of the HEBM-milled PC presented here also provides these benefits. Beyond the scope of this work, which focuses on ultrafast dispersion by milling and particle size measurement of the RLS precursor, these aspects will be the subject of our future investigations.

The uniformity of the PC layer sets the basis for reproducible Cu layers, such as traces or sensors. As described by Binh Nam et al. [37], the generation of small and highly dispersed CuO NPs reduces the surface roughness of the Cu structures, which is supposed to counteract oxidation. In addition, the edge regions of individual Cu lines become more homogeneous and planar layers acquire a metallic luster. The superiority of the prepared particles over commercially available powdered NPs has been demonstrated. High surface roughness resulting from too-large CuO particles can have a negative effect on the oxidation resistance of the resulting Cu layer. Small particles are more densely packed in the dry PC layer, they are more reactive and they have a high plasmon resonance and a lower melting point after reduction. Based on the results shown here, it must be concluded that ultrasonic dispersion is not sufficient, which results in this superiority, but also that commercial powders can still be used, by employing HEBM.

## 4. Conclusions

In conclusion, this work demonstrated and comprehensively discussed the possibility of producing highly filled and finely dispersed copper(II) oxide nanosuspensions as a precursor for femtosecond-laser reductive laser sintering by high-energy ball milling. The comparison to an undispersed precursor showed the compelling need for sufficiently high dispersion and the absence of large agglomerates; otherwise, defects are created in the Cu structure. These defects have the potential to completely disrupt an electrical circuit. The resulting long-term stable nanofluid exhibited an advantageous trimodal particle size distribution, with percentiles D10: 18 nm, D50: 50 nm and D90: 560 nm, resulting in dry PC layers of the highest homogeneity and a low surface roughness of 88 nm. This homogeneity provided the basis for the production of uniform and reproducible copper layers for the electrification of polymer substrates with an excellent sheet resistivity of 0.278 Ω/sq and an elemenatal copper mass content of 94.2%. The study of the undispersed PC and the resulting Cu structures showed the need for high dispersity and freedom from agglomerates. In addition, the superiority of this method over ultrasonic dispersion, which has been established by RLS research to date, was demonstrated in a comparative study. The latter method still contained agglomerated particles in a size range from 750 nm to 12 μm, and it was therefore unsuitable for reproducible RLS. In the context of particle size measurement, we showed that the favorable measurement method of laser diffraction in combination with dynamic image analysis can be used to characterize the dispersion process, but also represents a high sensitivity to oversized particles contained in the suspension.

## Data Availability

Not applicable.

## Abbreviations

The following abbreviations are used in this manuscript:

Copper	Cu
Copper(II)-oxide	CuO
HEBM	high-energy ball milling
LSM	laser scanning microscopy
NPs	nanoparticles
PC	precursor
RLS	reductive laser sintering
SEM	scanning electron microscopy
UD	undispersed
US	ultrasonic dispersion
